# MobileNet-V2 /IFHO model for Accurate Detection of early-stage diabetic retinopathy

**DOI:** 10.1016/j.heliyon.2024.e37293

**Published:** 2024-08-31

**Authors:** Chunjuan Huang, Mohammad Sarabi, Adham E. Ragab

**Affiliations:** a Guangling College, Yangzhou University, Yangzhou, China; b Ankara Yıldırım Beyazıt University (AYBU), 06010, Ankara, Turkey; c Industrial Engineering Department, College of Engineering, King Saud University, PO Box 800, Riyadh 11421, Saudi Arabia

**Keywords:** *Diabetic retinopathy*, *Automated image processing algorithms*, *Mobilenet-V2*, *Improved fire hawk optimizer*, *Early-stage detection model*

## Abstract

Diabetic retinopathy is a serious eye disease that may lead to loss of vision if it is not treated. Early detection is crucial in preventing further vision impairment and enabling timely interventions. Despite notable advancements in AI-based methods for detecting diabetic retinopathy, researchers are still striving to enhance the efficiency of these techniques. Therefore, obtaining an efficient technique in this field is essential. In this research, a new strategy has been proposed to improve the detection of diabetic retinopathy by increasing the accuracy of diagnosis and identifying cases in the initial stages. To achieve this, it has been proposed to integrate the MobileNet-V2 deep learning-based neural network with Improved Fire Hawk Optimizer (IFHO). The MobileNet-V2 network has been renowned for its efficiency and accuracy in image classification tasks, making it a suitable candidate for diabetic retinopathy detection. By combining it with the IFHO, the feature selection process has been optimized, which is essential for identifying relevant patterns and abnormalities related to diabetic retinopathy. The Diabetic Retinopathy 2015 dataset has been used to evaluate the effectiveness of the MobileNet-V2/IFHO model. The study results indicate that the DRMNV2/IFHO model consistently outperforms other methods in terms of precision, accuracy, and recall. Specifically, the model achieves an average precision of 97.521 %, accuracy of 96.986 %, and recall of 98.543 %. Moreover, when compared to advanced techniques, the DRMNV2/IFHO model demonstrates superior performance in specificity, F1-score, and AUC, with average values of 97.233 %, 93.8 %, and 0.927, respectively. These results underscore the potential of the DRMNV2/IFHO model as a valuable tool for improving the accuracy and efficiency of DR diagnosis. Nevertheless, additional validation and testing on larger datasets are required to verify the model's effectiveness and robustness in real-world clinical scenarios.

**Table:** 

Nomenclature		Abbreviations	
**A(D)**	Fitness function	AUC	Area Under The Curve
**B**	Batch size	CNN	Convolutional Neural Network
**d**	Certain problem's dimension	DR	Diabetic Retinopathy
**f(a)**	An adaptive function collection and emission of combusting twigs)	FSL	Few-Shot Learning
**FN**	False Negative	GA	Generic Algorithm
**FP**	False Positive	GOA	Grasshopper Optimization Algorithm
**H**_**near**_	A different Fire Hawk in the search space	HHO	Harris Hawks Optimization
$H_{i}^{new}$	New situation of the l-th Fire Hawk	IFHO	Improved Fire Hawk Optimizer
**k**th	Prey out situation	JA	Jaya Algorithm
**LR**	Learning rate	MBO	Monarch Butterfly Optimization
**l**th	Fire hawk situation	MFO	Moth Flame Optimization
**m**	Total quantity of validation examples	ML-FEC	Multi-Label Feature Extraction and Classification
**N**	Number of neurons in the hidden layer	MLLD	Mining Local And Long-Range Dependence
**n**	Number of decision variables	MNV2	Mobile/NetV2
**P**_**q**_	Prey enclosed	OCT	Optical Coherence Tomography
$p_{q}^{new}$	Vector representing the new encircled's situation	ReLU	Layer, Batch Normalization And Rectified Linear Unit
**rand**	Uniformly spread stochastic amount	SMOTE	Synthetic Minority Oversampling Technique
**S**	Search space	SSA	Salp Swarm Algorithm
**S**_**l**_	Secured locations under the Fire Hawk region	SVM	Support Vector Machine
**TN**	True Negative	WM	Wang-Mendel
**TP**	True Positive		
**Z**_**i**_	Candidate solution in the search space		
$Z_{i}^{j}\left(0\right)$	Candidate solution's first situation		

## 1 Introduction

A condition that affects the ocular system, diabetic retinopathy is a complication of diabetes. The photosensitive tissue located at the posterior part of the eye, which is the retina, becomes affected due to the impairment of the blood vessels. Two primary forms of diabetic retinopathy exist, namely non-proliferative and proliferative. In the initial stage, minute blood vessels within the retina experience leakage of fluid or blood, leading to the formation of deposits known as “exudates”. Consequently, the macula, responsible for acute vision, may undergo swelling. Symptoms may manifest as blurred vision, floaters, and color perception difficulties [1]. As the disease progresses, the retina's oxygen supply diminishes, prompting the growth of abnormal blood vessels. These fragile vessels can rupture within the vitreous gel, resulting in visual impairments, such as floaters, shadows, or even sudden vision loss [2]. Furthermore, the abnormal blood vessels can generate scar tissue, which may exert traction on the retina, ultimately leading to retinal detachment [3]. The progression of diabetic retinopathy is influenced by various factors like the length of time a person has had diabetes, suboptimal management of blood sugar levels, raised blood pressure, increased levels of cholesterol, and pregnancy. Regular ocular examinations play a pivotal role in the identification and monitoring of diabetic retinopathy [4]. Ophthalmologists employ various diagnostic techniques, such as dilated eye examinations, retinal photography, or Optical Coherence Tomography (OCT), to assess the extent of retinal damage [5].

Diabetic retinopathy can be identified through a comprehensive ocular examination. In the course of a dilated eye examination, a specialist in ophthalmology or optometry will use specialized eye drops to clarify the issue to the pupils, thereby facilitating a more thorough examination of the retina and its vasculature. Subsequently, the physician will scrutinize the retina using a variety of instruments and methodologies to identify any indications of diabetic retinopathy [6]. Common diagnostic procedures encompass a visual acuity assessment to gauge the acuteness of vision, fundus photography to capture high-resolution images of the retina, fluorescein angiography to monitor blood circulation and identify anomalous vessels, OCT to furnish comprehensive data regarding retinal thickness and integrity, and tonometry to measure intraocular pressure [7]. Regular ocular examinations are of paramount importance for individuals afflicted with diabetes, as early detection and intervention can effectively manage diabetic retinopathy and prevent potential vision impairment.

Diabetic retinopathy can be diagnosed with the utilization of artificial intelligence techniques, specifically image processing and neural networks. These methodologies enhance the computational power of algorithms to examine retinal images and identify indications of diabetic retinopathy. Image processing algorithms are employed to enhance and segment retinal images, facilitating the detection of abnormalities. This includes contrast enhancement, noise removal, and the highlight of specific features of interest. This preprocessing stage prepares the images for subsequent analysis. Subsequently, neural networks, a form of machine learning algorithm [8], are applied to the preprocessed images [9]. These networks are trained on extensive datasets of retinal images, which possess known labels indicating the presence or absence of diabetic retinopathy. By learning from these labeled datasets, neural networks acquire the capability to classify new, unseen retinal images based on their distinctive features and patterns.

The diagnosis of diabetic retinopathy through retinal image analysis involves assessing different characteristics, such as the shape, size, distribution of blood vessels, the presence of hemorrhages or exudates, and the condition of the macula, using a neural network. The neural network compares these features to patterns observed in the training data to make a diagnosis. To ensure the accuracy and reliability of the AI diagnosis, these algorithms are typically validated against a large number of retinal images, including both normal and diseased cases, and compared to the diagnoses made by human experts. This process helps to refine the algorithms and improve their performance.

The diagnosis of diabetic retinopathy has shown promising results through the application of AI-based techniques, which can aid healthcare professionals in screening and early detection. They can also aid in prioritizing patients for further evaluation by ophthalmologists, particularly in regions with limited access to eye care specialists. However, it is important to note that AI-based diagnosis should always be validated and used in conjunction with clinical judgment by trained medical professionals.

## 2 Related works

Various works were done in this context. For example, it was reported that optimization algorithms can be an accurate and robust approach to solve second-order value problems [10,11]. Murugappan et al. [12] conducted the challenges associated with training models using small datasets by proposing a paradigm known as Few-Shot Learning (FSL). The researchers introduced a novel prototype network, DRNet, which utilizes attention-based grading and detection for Diabetic Retinopathy (DR). Episodic learning was utilized to train the DRNet framework on few-shot classification tasks, using the APTOS2019 dataset. The attention mechanism of the network was developed through aggregated transformations and gradient activations of classes to capture image representations. The model displayed impressive results, with high sensitivities, specificities, and accuracies in detecting and grading DR. Objective performance metrics and model interpretation revealed that the proposed model outperformed existing methods when handling previously unseen fundus images, making it a valuable tool for providing a second opinion on the severity level of DR. It is worth mentioning that the use of the APTOS2019 dataset in this study might have certain limitations that could potentially introduce biases or constraints when applied to other datasets or real-life situations. Additionally, the study did not explore potential challenges or limitations related to the scalability or generalizability of the proposed model beyond the specific context of DR detection and grading.

A method for identifying DR was proposed by Usman et al. [13] in their research paper. Their proposed model, called Deep Learning Multi-Label Feature Extraction and Classification (ML-FEC), is based on pre-trained Convolutional Neural Networks (CNNs) architecture. The researchers performed data preprocessing and feature extraction on the color Fundus Photographs using Principal Component Analysis before training a subset of images, using three CNN architectures through transfer learning. The proposed model has proven its effectiveness with accuracy rates ranging from 91.94 % to 94.40 % in the experimental results. This made it a suitable option for implementing in daily clinical practice and to support large-scale DR screening programs. However, it is worth noting that the evaluation was limited to a specific dataset, and its performance might differ when applied to different datasets or real-world scenarios. Additionally, the study did not consider the interpretability of the model, which is crucial for clinicians to comprehend the reasoning behind the prediction and treatment decisions.

Luo et al. [14] identified a limitation in current automatic DR detection methods that primarily relied on local information and failed to consider the long-distance dependencies between lesion features scattered throughout retinal fundus photographs. To address this constraint, a technique was suggested by the scientists that integrated associations among extensive patches into the deep learning structure. The proposed method enhanced the local patch features by utilizing patch-wise relationships, particularly for plaque-like lesions associated with DR. The implementation of the Long-Range unit contributed to the attainment of this goal. This unit had a residual structure that could be easily integrated into other trained networks. Several experiments were carried out to evaluate the efficacy of this approach, utilizing the Messidor and Eye-PACS datasets. The obtained results indicated that the proposed method was superior to the existing state-of-the-art models. Nevertheless, it is essential to recognize the limitations of this research. Firstly, the evaluation was conducted on specific datasets, which might not fully represent the diversity of real-world clinical settings. Therefore, the generalizability of the proposed method in different clinical scenarios should be further investigated. Additionally, the study did not address potential challenges related to the interpretability of the proposed method or its ability to be generalized to different severity levels of DR or other ocular diseases. These aspects should be considered in future research to ensure the practical applicability of the proposed approach.

Das et al. [15] developed an automated system for DR detection from retinal fundus images. The optimal parameters for the CNN model in diabetic retinopathy classification were automatically determined using a genetic algorithm-based technique proposed by the researchers. The CNN architecture comprised convolution and pooling layers for feature extraction, followed by a Support Vector Machine (SVM) for classification. By applying the proposed method to the Messidor dataset, the researchers achieved a superior performance compared to existing methods, with an accuracy of 0.9867 and an AUC of 0.9933. It is crucial to recognize the restrictions of this research, which encompassed the inadequate assessment of just one dataset. This limitation could potentially hinder the applicability of the suggested approach to other datasets or clinical settings in the real world. Furthermore, the study did not investigate the interpretability or robustness of the automated CNN design in handling variations in image quality and different stages of diabetic retinopathy.

Tagmatova et al. [16] developed a model for generating synthetic medical data to forecast diabetes type 2 based on artificial intelligence algorithms. The prediction accuracy of that model was over 94 % during neural network training of the dataset. Mutawa et al. [17] developed the dataset combination and model performance for diabetic retinopathy detection based on the Visual Geometry Group, Inception version 3, Dense Network, and Mobile Network version 2. That model could help workers refer patients to ophthalmologists before diabetic becomes serious. Omar and El-Hafeez [18] used deep learning models for epileptic seizure detection under feature scaling and dropout layers. Eliwa et al. [19] developed an approach employing a convolutional neural network to classify monkeypox skin lesions. Furthermore, they enhanced the convolutional neural network architecture by employing the grey wolf optimizer. That led to a notable enhancement in the model's accuracy, precision, recall, F1-score, and AUC when compared to the non-optimized version. Hady and El-Hafeez [20] utilized a machine learning algorithm to forecast the pelvic tilt and lumbar angle in women experiencing urinary incontinence and sexual dysfunction.

Hassan et al. [21] explored the application of language models and deep learning methods in the automation of disease prediction based on symptoms within the framework of Medical Concept Normalization and a Bidirectional Long Short-Term Memory model. In a real-world case study conducted in Egypt, Farghaly et al. [22] applied a machine learning framework to predict the Hepatitis C Virus. The researchers found that the random forest classifier was able to achieve an accuracy of 94.1 % with a learning elapsed time of only 0.55 s. The value of a machine learning-based framework to uncover more effective multidrug regimens for FDA-approved cancer treatment was reported [23]. It was also reported that machine-learning techniques could be used to optimize DNA sequence classification [24]. Furthermore, the utilization of machine learning and deep learning models has demonstrated remarkable precision in forecasting alterations in the core muscles amidst cases of female sexual dysfunction [25].

Bilal et al. [26] focused on the automation of DR diagnosis through the use of a two-stage approach. To detect diabetic retinopathy, a two-stage process was employed. In the first stage, optic disc and blood vessel segmentation were accomplished using independent U-Net models that incorporated preprocessing and data augmentation techniques to improve the quality of the image. The second stage utilized a hybrid CNN-SVD model that recognized biomarkers, such as microaneurysms, hemorrhages, and exudates, and incorporated transfer learning, which was capable of detecting DR. The suggested approach demonstrated exceptional results on EyePACS-1, Messidor-2, and DIARETDB0 datasets, with mean accuracies of 97.92 %, 94.59 %, and 93.52 %, respectively, outperforming previous methods. However, limitations may include the lack of evaluation of additional datasets, potential challenges in generalizing the approach to diverse populations, and the need for further validation in real-world clinical settings.

Diabetic retinopathy is a serious eye disease that may lead to loss of vision if it is not treated on time. Early detection is crucial in preventing further vision impairment and enabling timely interventions. Despite notable advancements in AI-based methods for detecting diabetic retinopathy, researchers are still striving to enhance the efficiency of these techniques. Therefore, obtaining an efficient technique in this field is essential. In pursuit of this goal, this paper introduces an innovative approach that combines the MobileNet-V2 neural network with an Improved Version of Fire Hawk Optimizer to improve the effectiveness of diabetic retinopathy detection. The MobileNet-V2 neural network is a widely used CNN architecture known for its compact and efficient design, making it suitable for image classification and object detection tasks.

The Improved Version of Fire Hawk Optimizer is a newly developed optimization algorithm that is based on the Firefly Algorithm. This algorithm has shown the ability to improve the convergence rate and generalization capability of deep learning models. The proposed method combines the strengths of the MobileNet-V2 and the Improved Version of the Fire Hawk optimizer, specifically to enhance the accuracy and efficiency of diabetic retinopathy detection. The MobileNet-V2 CNN is used to extract relevant features from retinal images, while the Improved Version of Fire Hawk Optimizer is used to optimize the classification process and improve the accuracy of the final diagnosis. This approach offers several potential benefits. Firstly, the use of the MobileNet-V2 CNN enables efficient processing of large datasets, thereby reducing the computational requirements and processing time necessary for accurate diagnosis. The Fire Hawk Optimizer's Improved Version also assists in enhancing the classification process of the model, ensuring precise identification and classification of various stages of diabetic retinopathy. Lastly, the proposed approach has the potential to seamlessly be employed in routine clinical screenings and examinations, enabling clinicians to make timely and accurate diagnoses. Therefore, the specific goals and contributions of the current paper are as follows:

• Developing a New Approach for Accurate Detection of Early-Stage Diabetic Retinopathy Based on a hybrid optimization model;
• Improving the effectiveness of diabetic retinopathy detection and the convergence rate and generalization capability of deep learning models by combining the Mobilenet-V2 neural network with an Improved Fire Hawk Optimizer;
• Reducing the computational requirements and processing time necessary for accurate diagnosis due to the efficient processing of large datasets;
• Enhancing the classification process of the model, ensuring precise identification and classification of various stages of diabetic retinopathy;
• Achieving a high-potential model for being employed in routine clinical screenings and examinations, enables clinicians to make timely and accurate diagnoses.

## 3 Methodology

**Fig. 1:**
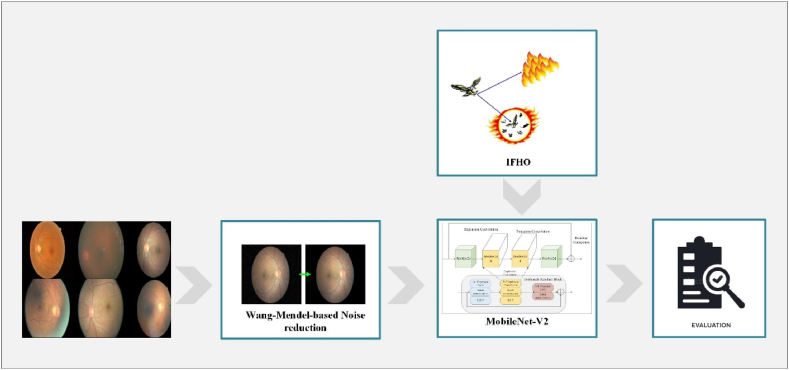
Block diagram of the proposed methodology.

This study introduces an innovative method for the precise identification of early-stage diabetic retinopathy through the utilization of advanced techniques. The initial step involves the utilization of the “Diabetic Retinopathy 2015 Data Colored and Resized” dataset to provide essential input data for the model. To enhance the quality of the input images, the Wang Mendel-based noise reduction technique is employed to reduce unwanted artifacts and enhance overall data clarity. Subsequently, the MobileNet-V2 architecture is utilized as a robust feature extractor on the preprocessed images for diabetic retinopathy diagnosis. This deep learning model is proficient in capturing intricate disease-related patterns and features. Fig. (1) displays the block diagram of the proposed methodology.

To further enhance the performance of MobileNet-V2, an improved version of the Fire Hawk optimizer (IFHO) is introduced. The IFHO algorithm improves the model's convergence and generalization capabilities, resulting in more accurate and robust predictions. By combining IFHO with MobileNet-V2, a powerful and adaptable system is created that can effectively learn from complex data distributions.

A comprehensive evaluation is conducted to assess the efficacy of the proposed approach, utilizing various performance metrics such as precision, specificity, accuracy, sensitivity, F1-score, and AUC.

## 4 Dataset description

The “Diabetic Retinopathy 2015 Data Colored and Resized” was the dataset employed in this research. This section presents a complete representation of the dataset and its significance within the context of the research investigation. The dataset encompasses retinal images that have undergone resizing and colorization procedures, making them suitable for diverse image analysis and machine-learning applications about this specific medical disease.

**Fig. 2:**
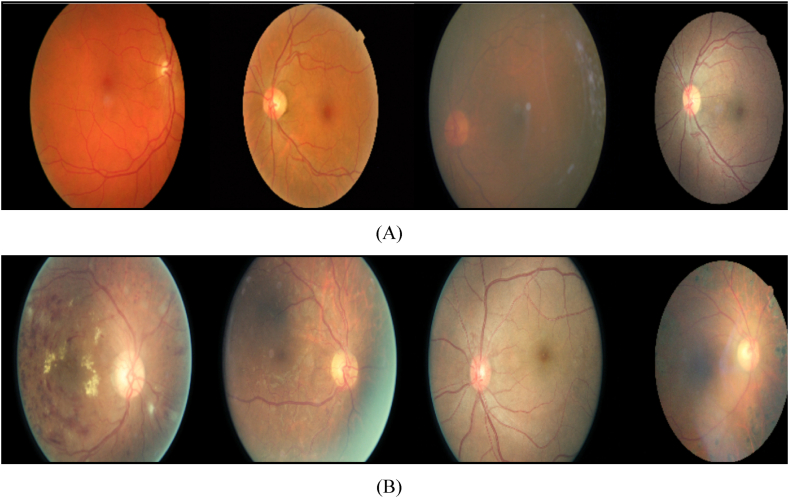
Examples of the dataset under investigation, with (A) representing images without Diabetic Retinopathy (No-DR), and (B) representing images with Diabetic Retinopathy (DR).

Primarily, the collection comprises retinal images obtained through fundus photography, a technique that captures the posterior segment of the eye, including the blood vessels, optic nerve, and retina. The provided images are obtained from individuals diagnosed with diabetic retinopathy. These images have been systematically organized to enhance their practicality and accessibility for various purposes. Fig. (1) showcases a selection of samples from the dataset under scrutiny. It can be seen from Fig. (2) that there are some examples of the dataset under investigation, representing images without Diabetic Retinopathy (No DR), and representing images with Diabetic Retinopathy (DR).

The pictures utilized in this research were acquired from the Kaggle competition focused on Diabetic Retinopathy. Each image has been assigned a label that corresponds to one of five abnormality states commonly associated with diabetic patients, namely “No-DR”, “Mild”, “Moderate”, “Proliferative DR”, and “Severe”.

To enhance their practicality in conjunction with a range of pre-trained deep learning models, these images have been resized to dimensions of 224×224 pixels. The training set consists of 35,126 resized and cropped images, while the test set contains 53,576 resized and cropped images. This adjustment proves advantageous when employing machine learning algorithms that require uniform input sizes. The dataset comprises a comprehensive collection of retinal images, each of which is allocated to a detailed grade that corresponds to the severity of diabetic retinopathy. These severity levels range from mild to severe, demonstrating the progression of the disease. This classification system enables researchers to analyze the patterns and characteristics associated with distinct stages of diabetic retinopathy.

The collection contains retinal photos that have been both colorized and scaled, thereby assuring uniformity and promoting streamlined processing. The technique of scaling photographs preserves the original aspect ratio while decreasing their total size. This not only improves computing efficiency but also facilitates wider accessibility. The offered dataset has potential use for diverse research endeavors in the field of diabetic retinopathy. The dataset is readily accessible on the Kaggle platform, enabling academics, physicians, and developers to get and use it for their inquiries. The dataset under analysis is available for access on [27].

## 5 Preprocessing

### 5.1 Wang-Mendel-based noise reduction

Wang-Mendel-based noise reduction is a signal-processing technique widely employed in the field of image processing to eliminate noise from signals. This technique, named after its proponents Wenwu Wang and Jerry Mendel [28], relies on the utilization of fuzzy logic systems to effectively capture and model inherent uncertainty in the input data.

The fundamental principle underlying the Wang-Mendel (WM) algorithm involves the conversion of noisy data into a fuzzy domain. Within this domain, membership functions are employed to represent the extent to which each pixel belongs to distinct fuzzy sets. These sets correspond to varying levels of noise and signal strength, thereby enabling the algorithm to differentiate between the two.

Once the data is transformed into the fuzzy domain, the noise is eliminated through the utilization of adaptation techniques that adjust the membership functions of the pixels. Essentially, the algorithm identifies pixels that do not possess high membership in any set and modifies their membership functions to incorporate them into a set that exhibits a stronger representation of the signal [29]. The outcome of this process is an image that is clear of a significant portion of noise. A variety of applications, such as medical imaging, have provided evidence of the effectiveness of the WM-based approach.

**Table:** 

Algorithm 1: Wang-Mendel (WM) algorithm for Diabetic Retinopathy image
Input: Diabetic Retinopathy image with noise Output: Cleaned-up Diabetic Retinopathy image 1. Define membership functions and fuzzy sets: - Define fuzzy sets that represent different levels of noise and signal strength. - Define membership functions for each fuzzy set. 2. Transform the noisy image into the fuzzy domain: - Convert each pixel value of the input image into fuzzy membership values using the defined membership functions. 3. Apply noise removal using adaptation techniques: - For every pixel in the fuzzy domain, calculate the membership values for each fuzzy set. - Identify pixels that do not belong to any set with high membership. - Adjust the membership functions of these pixels to include them in a set with a stronger signal representation. - Iterate this process until the desired noise reduction is achieved. 4. Transform the cleaned-up fuzzy image back to the spatial domain: - Convert the fuzzy membership values of each pixel back into corresponding spatial pixel values. 5. Return the cleaned-up Diabetic Retinopathy image.

**Fig. 3:**
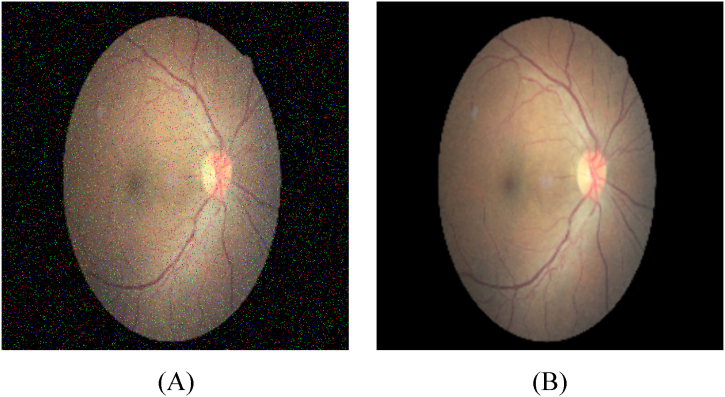
Wang-Mendel-based noise reduction, with (A) representing the original image and (B) showcasing the resulting denoised image.

Fig. (3 (A, B)) illustrates a sample of the Wang-Mendel-based noise reduction technique applied to a Diabetic Retinopathy image.

It can be seen from Fig. (2-A) that the unprocessed original image is depicted before the application of any noise reduction procedure. This image may contain a 0.04 salt and pepper noise in pixel values or distortions caused by imaging artifacts. These noise elements can impair the quality and accuracy of image analysis and interpretation.

However, Fig. (2-B) displays the denoised image obtained through the utilization of the Wang-Mendel-based noise reduction algorithm. By employing fuzzy logic principles and adaptation techniques, this algorithm effectively discerns and differentiates between noise and signal components within the image. To decrease the noise while maintaining the crucial features and structures of the underlying Diabetic Retinopathy image, a sequence of modifications and adaptations of membership functions are employed.

### 5.2 Synthetic Minority Oversampling Technique (SMOTE)

The issue of class imbalance in datasets is prevalent in the fields of machine learning and statistical modeling. It occurs when the number of examples of one class is significantly smaller than another. To address this, the Synthetic Minority Oversampling Technique (SMOTE) is a popular algorithmic approach employed. It effectively tackles the problem of class imbalance in datasets and is widely used. The accurate handling of class imbalance is crucial for the performance and accuracy of models, particularly in cases where the minority class is of significant interest.

Generating synthetic data samples for the minority class is the main goal of the SMOTE algorithm to overcome class imbalance. Instead of oversampling with replacement, SMOTE creates synthetic instances of the minority class by interpolating between existing minority class examples. To achieve this, the algorithm chooses a minority class instance and identifies its k-nearest neighbors. A new sample is generated by selecting one of the nearest neighbors randomly and calculating the difference between the selected instance and this neighbor, which is also chosen by the algorithm randomly. The difference is then multiplied by a random number between 0 and 1, and added to the original instance to create a new synthetic instance.

**Table:** 

Algorithm 2: SMOTE algorithm for Diabetic Retinopathy image augmentation
input: minority_samples - list of minority class images k - number of nearest neighbors to consider synthetic_sample_count - desired number of synthetic samples to generate output: synthetic_samples - list of synthetic samples generated by SMOTE function generate_synthetic_sample(minority_sample, nearest_neighbor): synthetic_sample = () for pixel in range(number_of_pixels): difference = nearest_neighbor(pixel) - minority_sample(pixel) random_number = generate_random_number_between_0_and_1() synthetic_value = minority_sample(pixel) + difference * random_number synthetic_sample.append(synthetic_value) return synthetic_sample function smote(minority_samples, k, synthetic_sample_count): synthetic_samples = () while synthetic_sample_count >0: minority_sample = randomly_select_sample(minority_samples) nearest_neighbors = find_k_nearest_neighbors(minority_sample, minority_samples, k) nearest_neighbor = randomly_select_nearest_neighbor(nearest_neighbors) synthetic_sample = generate_synthetic_sample(minority_sample, nearest_neighbor) synthetic_samples.append(synthetic_sample) synthetic_sample_count - = 1 return synthetic_samples

As mentioned before, there are 88,702 resized and cropped images that increased by 100,000 after applying the SMOTE algorithm.

## 6 Modified MobileNet-V2

### 6.1 MobileNetV2

Deep learning is a subset of artificial intelligence that utilizes neural network structures with multiple layers to withdraw input data progressively [30]. With the aid of powerful computational capabilities and extensive databases, deep learning models excel in complex pattern recognition tasks that surpass human capabilities, such as computer vision, natural language processing, and speech synthesis [31]. In the realm of mobile applications, MobileNetV2 is a highly regarded deep learning architecture designed to enhance computation speed and reduce memory usage, specifically tailored for edge devices. MobileNetV2 achieves remarkable efficiency by employing depthwise separable convolutions and residual connections, enabling exceptional performance even in resource-constrained environments. As a result, developers are increasingly integrating MobileNetV2 into various mobile-based AI applications, ranging from facial recognition to autonomous navigation systems. This exemplifies the expanding scope of deep learning in today's digital era [32].

MobileNetV2's main characteristic is the implementation of a lightweight bottleneck architecture, which substantially decreases the amount of parameters and computation needed when compared to conventional convolutional neural networks; however, it retains high accuracy. This bottleneck architecture involves a process whereby the spatial dimensions of the input are first reduced using a 1×1 convolution (known as the “expansion layer”), followed by a depthwise convolution operation to extract features, and another 1×1 convolution (known as the “projection layer”) is used to expand the feature maps back out. The architecture of MobileNetV2 can be categorized into three primary constituents.

The first component is the stem block, which is responsible for the initial processing of the input image. After a 3×3 Convolutional layer, Batch Normalization and Rectified Linear Unit (ReLU) activation follow.

The second component, known as the MobileNetV2 body, is composed of multiple stacked residual bottleneck blocks. Each block consists of a sequence of 1×1 and 3×3 convolutions that ReLU and Batch Normalization have been applied after each convolution. Additionally, a shortcut connection is involved that bypasses the convolutional layers and adds the input to the output of the convolution sequence.

**Fig. 4:**
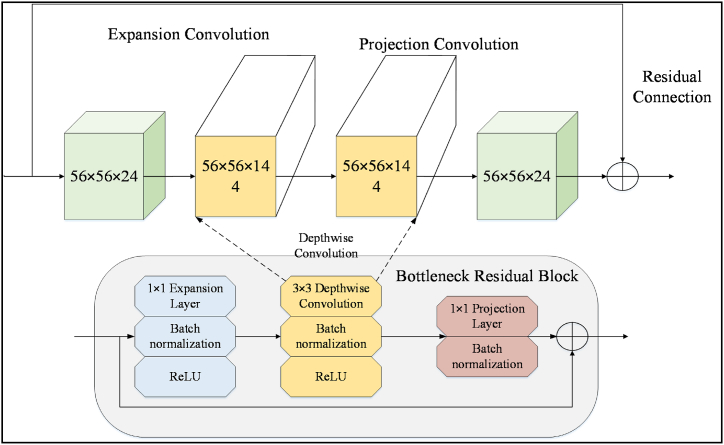
Architecture of the MobileNetV2.

The dimensionality of the feature maps gradually rises and falls in the network, with the number of channels changing accordingly. In the classification head, the last bottleneck block's feature maps are employed in a single vector, using a global average pooling layer. A fully connected layer with softmax activation follows, generating a probability score for each class. A diagram illustrating the structure of a typical bottleneck block in MobileNetV2 is presented below. Fig. (4) illustrates the architecture of MobileNetV2.

The architectural design commences with a depthwise separable convolutional layer, which performs spatial convolutions on each input channel individually. Afterward, a layer of batch normalization is added, followed by the application of a ReLU activation function. The resulting output is then fed into a bottleneck layer, which utilizes a 1×1 convolution to decrease the number of filters. This is followed by a nonlinear activation function and another 1×1 convolution to expand the feature maps once again.

Moreover, the MobileNetV2 architecture incorporates residual connections between the bottleneck layers. These connections facilitate the smooth flow of gradients throughout the network, thereby enhancing the training process and accuracy. Additionally, the architecture employs a technique known as inverted residuals, wherein the bottleneck layer is applied to an expanded input. This technique effectively enhances the model's representation power.

### 6.2 MobileNetV2 for DR diagnosis (DRMNV2)

To utilize the MobileNetV2 to diagnose Diabetic Retinopathy (DR) images, the following procedures have been taken into account.

(1) A collection of DR images has been compiled with corresponding diagnosis labels indicating the degree of DR severity, ranging from healthy to various stages of the condition. The dataset has been split into two sets with the training set constituting 80 % and the test set 20 %. Then, data preparation has been completed.
(2) Input Preprocessing: The images have been resized to a consistent size suitable for MobileNetV2 input, for example, 224×224 pixels. Additionally, the values of pixels have been normalized to fall within the range [0, 1] by using the min/max normalization.
(3) The training set: It was used to train the MobileNetV2 model. While training, the aim was to optimize the model's parameters, including weights and biases, to minimize the difference between predicted labels and true labels. Techniques like stochastic gradient descent or its variations are typically employed to achieve this. The choice of loss function is determined by the specific problem formulation, such as binary cross-entropy for binary classification or categorical cross-entropy, for multi-class classification.
(4) Assessing the performance of the trained model: The trained model has been evaluated on the test set. For classification tasks, various evaluation metrics such as precision, sensitivity, specificity, accuracy, AUC, and F1 score are commonly used.

The model has been in the following to have a thorough understanding of it. Here, the input image has been regarded as x, and the output of each layer has been regarded as h_i, where i is the layer index.

**A. Stem Block**.

**Table:** 

h_0 = Convolution(x) h_1 = BatchNormalization(h_0) h_2 = ReLU(h_1)

**B. MobileNetV2 Body:** The body consists of multiple bottleneck blocks. Each block has three layers, comprising expansion convolution, depthwise convolution, and projection convolution.

**Table:** 

h_i = BottleneckLayer(h_i-1)

**C. The BottleneckLayer:** This layer has been clarified in the following.

**Table:** 

h_tmp = Convolution1x1(h_i-1) h_tmp = BatchNormalization(h_tmp) h_tmp = ReLU(h_tmp) h_tmp = DepthwiseConvolution3x3(h_tmp) h_tmp = BatchNormalization(h_tmp) h_tmp = ReLU(h_tmp) h_tmp = Convolution1x1(h_tmp) h_tmp = BatchNormalization(h_tmp) # Shortcut connection if stride = = 1 and input_channels = = output_channels: # If no downsampling occurs h_i = h_i-1 + h_tmp else: # Downsampling occurs h_i = h_tmp

**D. Classification Head:** Just like the previous layers, this layer also has been clearly expressed so that readers can understand it better.

**Table:** 

h_final = GlobalAveragePooling(h_i) output = FullyConnectedSoftmax(h_final)

The prior mathematical formulation denotes the forward pass of the MobileNetV2 architecture. During the training process, backpropagation is used to compute the loss function's gradients concerning the parameters, and the optimizer adjusts the parameters to reduce the loss. The precise particulars and hyperparameters are contingent upon the implementation and framework utilized for model training. Upon completion of the training process, when presented with a new DR image, the MobileNetV2 architecture can be utilized to derive the predicted diagnosis label using the aforementioned forward pass formulation.

A metaheuristic algorithm with some modifications has been used to effectively arrange the DRMNV2 in the present study.

### 6.3 Optimization of the DRMNV2

#### 6.3.1 Decision variables

The hyperparameters of a neural network are associated with the decision variables. In this specific case study, three hyperparameters have been considered, namely the number of neurons in the hidden layer (N), the learning rate (LR), and the batch size (B). Consequently, the number of decision variables equals n=3, and D=(N,LR,B).

#### 6.3.2 Search space

The search space encompasses the range of values that can be assigned to each decision variable. For every decision variable $d_{i}$, its search space can be denoted as $S_{i}=\left(l_{i},u_{i}\right)$, where $l_{i}$ represents the lower bound, and $u_{i}$ represents the upper bound. Here, the following search spaces are considered:

- Quantity of neurons in the hidden layer (N): $S_{1}=\left(50,200\right)$.
- Learning rate (LR): $S_{2}=\left(0.001,0.01\right)$.
- Batch size (B): $S_{3}=\left(32,128\right)$.

Consequently, the overall search space is determined by the Cartesian product of the individual search spaces: $S=S_{1}\times S_{2}\times S_{3}$.

#### 6.3.3 Fitness function

The neural network's effectiveness is evaluated using a particular set of hyperparameters called D, using a metric known as the fitness function. In this instance, the validation accuracy of the neural network can be employed as the fitness function. Thus, the fitness function F(D) corresponds to the validation accuracy achieved by training the neural network using the hyperparameters within the set D.

The expression of fitness function A(D) can be articulated subsequently [equation (1)].

(1) $$A\left(D\right)=\left(\frac{1}{m}\right)\times \sum_{i=1}^{m}\left({\hat{y}}_{i}=y_{i}\right)$$

where, m represents the total quantity of validation examples, the symbol Σ denotes the summation of the individual results for each sample i ranging from 1 to m, the expression $\left[{\hat{y}}_{i}=y_{i}\right]$ is used to verify whether the predicted output ${\hat{y}}_{i}$ corresponds to the true label $y_{i}$, and the Iverson bracket notation (.) yields a value of 1 if the condition is true (i.e., the predicted output matches the true label), otherwise, it is 0.

As the value of A(D) increases, the performance of the neural network on the validation dataset improves. The objective is to optimize the fitness function A(D) by exploring the most suitable combination of hyperparameters, resulting in a higher accuracy on the validation dataset.

In this study, a modified metaheuristic algorithm, called the Improved Version of Fire Hawk optimizer, has been utilized for this purpose which is explained in detail in the following section.

## 7 Improved fire hawk optimizer (IFHO)

The motivation model of the enhanced FHO algorithm and the mathematical model of the recommended meta-heuristic algorithm have been presented in this section.

### 7.1 Motivation

Fire as an impressive instrument to bridle and preserve the equilibrium of the local environment and land is applied by Native Australians and has been a portion of national and principled customs since many years ago. Frequently, fires intentionally commenced or those that can arise from natural causes like lightning strikes can be amplified by human activities and other contributing factors, thereby heightening the susceptibility of the local environment and wildlife. Additionally, the responsibility of spreading fires across the land falls upon whistling kites, black kites, and brown falcons, which has been considered a reason that has only been recently comprehended. These avian creatures, referred to as Fire Hawks, actively attempt to propagate fires by transporting burning branches using their beaks and talons, a phenomenon that is documented as a detrimental occurrence in the natural world. Birds use a unique technique to catch their prey where they pick up burning twigs and drop them in non-burning areas to start small fires and manage to capture their prey. These minor fires instill fear in the prey, such as rodents, snakes, and other creatures, compelling them to hastily and anxiously escape which significantly facilitates the hawks' hunting efforts.

### 7.2 Mathematical model

By utilizing the technique of identifying and spreading fires and catching prey, the FHO meta-heuristic algorithm imitates the hunting behavior of fire hawks. The number of candidate solutions denoted as Z, is determined based on the position vectors of the fire hawks and prey. To start the search, these vectors are assigned initial positions in the search space using a random initialization process [equations (2), (3)].

(2) $$Z={\left[\begin{array}{c} Z_{1}\\ Z_{2}\\ \vdots \\ Z_{i}\\ \vdots \\ Z_{N} \end{array}\right]}_{N\times m}=\left[\begin{array}{c} \begin{array}{cc} \;z_{1}^{1}\;z_{1}^{2} & \ldots \end{array}\;\begin{array}{ccc} z_{1}^{j} & \ldots & z_{1}^{d} \end{array}\\ \begin{array}{cc} \;z_{2}^{1}\;z_{2}^{2} & \ldots \end{array}\;\begin{array}{ccc} z_{2}^{j} & \ldots & z_{2}^{d} \end{array}\\ \begin{array}{cc} \vdots & \ddots \end{array}\;\begin{array}{ccc} \vdots & \ddots & \vdots \end{array}\\ \begin{array}{cc} \;z_{i}^{1}\;z_{i}^{2} & \ldots \end{array}\;\begin{array}{ccc} z_{i}^{j} & \ldots & z_{i}^{d} \end{array}\\ \begin{array}{cc} \vdots & \ddots \end{array}\;\begin{array}{ccc} \vdots & \ddots & \vdots \end{array}\\ \begin{array}{cc} \;z_{N}^{1}\;z_{N}^{2} & \ldots \end{array}\;\begin{array}{ccc} z_{N}^{j} & \ldots & z_{N}^{d} \end{array} \end{array}\right],\left\{ \begin{array}{c} i=1,2,\ldots .N.\\ j=1,2,3,\ldots ,d. \end{array}\right.\text{,}$$

(3) $$z_{i}^{j}\left(0\right)=z_{i,\min}^{j}+rand.\left(z_{i,\max}^{j}-z_{i,\min}^{j}\right),\left\{ \begin{array}{c} i=1,2,\ldots .N.\\ j=1,2,3,\ldots ,d. \end{array}\right.$$

Here, $Z_{i}$ signifies the $i^{th}$ candidate solution in the search space; d signifies the certain problem's dimension; N is the whole number of candidate solutions in the solution space; $z_{i}^{j}$ is the $j^{th}$ decision variable of the $i^{th}$ candidate solution; $z_{i}^{j}\left(0\right)$ signifies the candidate solutions' first situation; $z_{i,\min}^{j}$, and $z_{i,\max}^{j}$ are the lowest and the highest boundaries of the $j^{th}$ decision variable for the $i^{th}$ candidate solution; and rand is a uniformly spread stochastic amount that is between 0 and 1. The evaluation of cost function value for candidate solutions is the key factor in identifying the Fire Hawks within the solution space. Those candidate solutions with higher cost values are chosen as Fire Hawks, while the others are designated as prey.

The selected Fire Hawks are utilized to spread fires across the solution space to facilitate hunting. The best global solution is usually used as the initial fire source by the Fire Hawks to propagate fires across the natural search space. The mathematical models for these features are defined by the following formulas [equations (4), (5)]:

(4) $$P=\left[\begin{array}{c} P_{1}\\ P_{2}\\ \vdots \\ P_{k}\\ \vdots \\ P_{m} \end{array}\right],k=1,2,\ldots ,m\text{.}$$

(5) $$H=\left[\begin{array}{c} H_{1}\\ H_{2}\\ \vdots \\ H_{l}\\ \vdots \\ H_{n} \end{array}\right],l=1,2,\ldots ,n\text{.}$$

In the solution space $P_{k}$ refers to the $K^{th}$ prey out of a total of m preys, while $H_{l}$ refers to the $l^{th}$ fire hawk out of a total of n fire hawks. The algorithm then calculates the distance between each fire hawk and prey to determine which prey is closest to each bird. This allows for the productive regions of each bird to be identified. Notably, the prey closest to the first fire hawk with the highest cost value is selected, while the remaining birds are assigned the remaining prey. The calculation is performed using the following formula [equation (6)]:

(6) $$D_{k}^{l}=\sqrt{{\left(z_{2}-z_{1}\right)}^{2}+{\left(y_{2}-y_{1}\right)}^{2}},\left\{ \begin{array}{c} l=1,2,3,\ldots ,n\\ k=1,2,3,\ldots .m \end{array}\right.$$

The distance between the $l^{th}$ fire hawk and the $k^{th}$ prey, denoted as $D_{K}^{l}$, is computed using a specific formula. The total number of prey and fire hawks in the solution space is represented by m and n, respectively. The coordinates of the Fire Hawks and prey are given by $z_{1}$ and $y_{1}$ as well as $z_{2}$ and $y_{2}$ within the solution space.

Once the overall distance between the Fire Hawks and their prey is calculated using the above method, the solution space for these birds is determined based on the nearest prey. The arrangement of the Fire Hawks and prey forms the basis of the algorithm's search process. It is important to note that the Fire Hawk with the highest cost value selects the closest prey in the solution space to occupy its specific region. Thus, the other Fire Hawks proceed to the next closest prey in the solution space, indicating that the stronger Fire Hawks are more effective hunters than their weaker counterparts.

During the next stage of the algorithm, the Fire Hawks collect burning twigs from the primary fire and transport them to the designated area. Each bird carries a burning twig and drops it in their assigned location to create a sense of urgency in the prey to flee rapidly. Meanwhile, some birds may collect burning twigs from other Fire Hawks' areas, and these two methods can be utilized as situation renewal processes in FHO's central search loop, as stated in the following formula:

(7) $$H_{l}^{new}=H_{l}+\left(r_{1}\times GB-r_{2}\times H_{near}\right),l=1,2,3,\ldots ,n$$

The vector represents the novel situation of the $l^{th}$ Fire Hawk is given by $H_{i}^{new}$. The solution space's central fire, referred to as GB, is the global best solution. A different Fire Hawk in the solution space is represented by $H_{near}$. To determine the movements of Fire Hawks towards the central fire and other Fire Hawks' regions, $r_{1}$ and $r_{2}$ have been employed, which are uniformly dispersed stochastic amounts ranging from 0 to 1.

During the next phase of the algorithm, the way the prey moves around each Fire Hawk is a crucial aspect of animal behavior that contributes to the situation-renewing process. When a Firehawk drops a burning twig, the prey can either hide, run away, or mistakenly move toward the Firehawk. These behaviors are quantifiable in the situation-renewing process using the following formula [equation (8)]:

(8) $$P_{q}^{new}=P_{q}+\left(r_{3}\times H_{l}-r_{4}\times S_{l}\right),\left\{ \begin{array}{c} l=1,2,3,\ldots ,n\\ q=1,2,3,\ldots .r \end{array}\right.$$

The vector represents the novel situation of the q encircled by the $l^{th}$ Fire Hawk $H_{i}^{q}$ is given by $p_{q}^{new}$. The global greatest solution in the solution space is represented by GB and acts as the central fire. Secured locations under the $l^{th}$ Fire Hawk region is denoted as ${\;S}_{l}$. The stochastic variables $r_{3}$ and $r_{4}$ are uniformly spread and are limited between 0 and 1 to specify the motions of prey toward the Fire Hawks and the secured location. Additionally, the prey may move toward other Fire Hawks' regions, and there is a possibility that they may become closer to the Fire Hawks during close ambushes or attempt to hide in a secured location outside the Fire Hawk's region if they are deceived. These activities can be measured in the situation renewal procedure using the following formula[equation (9)]:

(9) $$P_{q}^{new}=P_{q}+\left(r_{5}\times H_{Alter}-r_{6}\times S\right),\left\{ \begin{array}{c} l=1,2,3,\ldots ,n\\ q=1,2,3,\ldots .r \end{array}\right.$$

Here, $P_{q}^{new}$ is the novel situation vector of the $q^{th}$ prey $\left(P_{q}\right)$ surrounded by the $l^{th}$ fire hawk $\left(H_{l}^{q}\right)$; $H_{Alter}$ is another fire hawk in the solution space; S is a secured location outside the $l^{th}$ Fire Hawk's region. Uniformly spread stochastic amounts between 0 and 1, denoted as $r_{5}$ and $r_{6}$, are utilized to determine the movements of prey toward the additional Fire Hawks and the secure location outside the area. In reality, the secured location in the environment is a location where lots of creators collect to remain secure and sound while being in peril, the mathematic demonstration of $S_{l}$ and S is illustrated in the following[equation (10), (11)]:

(10) $$S_{l}=\frac{\sum_{q=1}^{r}P_{q}}{r},\left\{ \begin{array}{c} q=1,2,3,\ldots ,n\\ l=1,2,3,\ldots .r \end{array}\right.$$

(11) $$S=\frac{\sum_{k=1}^{m}P_{k}}{m},k=1,2,3,\ldots ,n$$

Here, $P_{q}$ is the $q^{th}$ prey surrounded by the $l^{th}$ fire hawk ($H_{l}$), and $P_{k}$ is the $k^{th}$ prey in the solution space.

It should be noted that each fire hawk's territory is considered a circular area. Therefore, the exact description of the territory depends on the distance between the prey and the fire hawk. In other words, if a prey is located within a particular fire hawk's territory, it is assumed that the prey might be affected by that specific fire hawk rather than others. Consequently, the number of prey and their proximity to the focal fire hawk determine the boundaries of their territory. During the situation renewal process, the likelihood of prey being outside their region is considered, as they may be influenced by fire hawks from other areas. The number of prey in each search cycle is determined by subtracting the number of fire hawks specified by a stochastic Gaussian spread, commonly used in randomization processes, from the total number of candidate solutions. The mathematical model of this algorithm takes into account the key features of FHO, such as the breach of candidate solutions' boundaries and the criteria for ending the process. In this context, a mathematical indicator is integrated into FHO, allowing for the management of each decision variable that violates boundaries. Termination criteria can be selected based on either a predetermined number of objective function evaluations or iterations, which are both provided as options.

### 7.3 Improved fire hawk optimizer (IFHO)

The Fire Hawk optimizer may necessitate modification to enhance its performance and augment its capacity to address optimization problems. Several potential rationales for modification encompass the following terms:

Constrained exploration capability: The initial algorithm may exhibit limitations in effectively exploring the search space, thereby yielding suboptimal solutions.

Sluggish convergence rate: The original algorithm may exhibit a slow rate of convergence, making it unsuitable for intricate optimization problems that demand swift convergence.

Untimely convergence: The original algorithm may become fascinated by local optima, resulting in premature convergence and obstructing its ability to ascertain the global optimum.

To enhance the optimization outcomes of the Improved Fire Hawk Optimizer, Eq. (7) can be modified subsequently [equation (12)]:

(12) $$H_{l}^{new}=H_{l}+\left(r_{1}\times GB-r_{2}\times H_{near}\right)\times f\left(\alpha \right)$$

where, f(α) is an adaptive function that modifies the impact of the collection and emission of combusting twigs. The parameter α serves as a control variable that can be dynamically updated throughout the optimization procedure.

The adaptive function f(α) can be expressed in the following manner [equation (13)]:

(13) $$f\left(\alpha \right)=\alpha +\left(1-\alpha \right)\times e^{-\gamma \times t}$$

where, the control parameter α governs the impact of the gathering and releasing burning twigs, with its value initially set to a small value and gradually increasing throughout the optimization process, the decay rate parameter γ regulates the rate at which α increases, and the variable t represents the current iteration or time step in the optimization process.

**Fig. 5:**
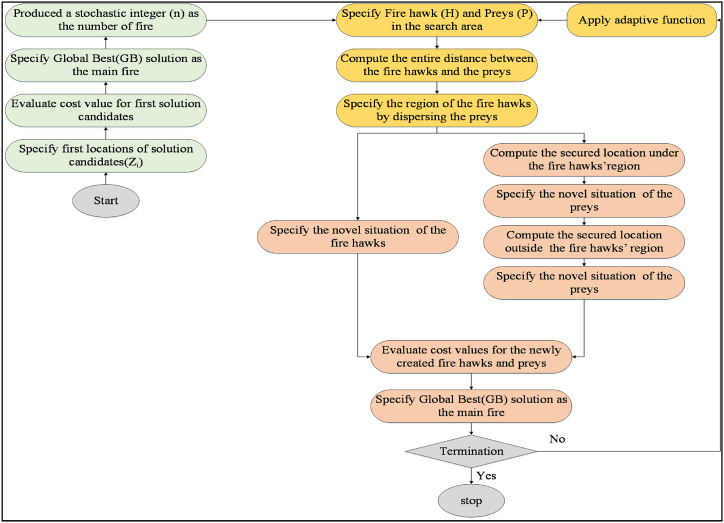
Flowchart diagram of the Improved Fire Hawk Optimizer.

By incorporating the adaptive function f(α), the algorithm is enabled to dynamically modify the influence of the gathering and releasing burning twigs, taking into account the present state of the optimization. This feature can enhance the algorithm's abilities in both exploration and exploitation, ultimately resulting in superior optimization outcomes. This Improved Fire Hawk optimizer's flowchart is illustrated in Fig. (5).

It should be noted that the MobileNet-V2 neural network is a widely used CNN architecture known for its compact and efficient design, making it suitable for image classification and object detection tasks. Besides that, the Improved Fire Hawk Optimizer is a newly developed optimization algorithm that is based on the Firefly Algorithm. This algorithm has shown the ability to improve the convergence rate and generalization capability of deep learning models. The proposed method combines the strengths of the MobileNet-V2 and Improved Fire Hawk Optimizer to enhance the accuracy and efficiency of diabetic retinopathy detection. The MobileNet-V2 CNN is used to extract relevant features from retinal images, while the Improved Fire Hawk Optimizer is used to optimize the classification process and improve the accuracy of the final diagnosis.

## 8 Results and discussions

The proposed model underwent validation by conducting experiments on standard practical datasets, with the outcomes being compared to those of some other contemporary methods. Details of the method validation have been explored in the subsequent subsections. The system used for the experiments was equipped with an Intel Core i7-10700K CPU, 16 GB DDR4 memory, and a graphics card of NVIDIA GeForce RTX 3070. For the Improved Fire Hawk Optimizer, the maximum number of generations, the maximum number of initial candidates, and the number of FireHawks are set to 1000, 25, and 200, respectively.

### 8.1 IFHO algorithm validation

This section aims to validate the Improved Fire Hawk Optimizer by conducting tests in a standardized benchmark test environment. To evaluate the effectiveness of IFHO in addressing optimization problems, it will be applied to the CEC-BC-2017 test case. The results will then be compared with six other metaheuristic algorithms that are currently considered state-of-the-art. These algorithms include the Salp Swarm Algorithm (SSA), Moth Flame Optimization (MFO) algorithm, Harris Hawks Optimization (HHO) algorithm, Grasshopper Optimization Algorithm (GOA), Jaya Algorithm (JA) [33], and Monarch Butterfly Optimization (MBO) algorithm. To ensure accuracy and reliability, all test functions were subjected to 30 runs for each algorithm. The algorithms utilized in this study are presented in Table 1 along with their corresponding parameter configurations.

**Table 1:** Parameter configurations used for the algorithms employed in this study.

Algorithm	Standard Values
Salp Swarm Algorithm (SSA)	N = 30, T = 100
Moth Flame Optimization (MFO) algorithm	N = 30, T = 100, b = 1
Harris Hawks Optimization (HHO) algorithm	N = 30, T = 100, E = 0.5
Grasshopper Optimization Algorithm (GOA)	N = 30, T = 100, c = 1
Jaya Algorithm (JA)	N = 30, T = 100
Monarch Butterfly Optimization (MBO) algorithm	N = 30, T = 100, Pp = 0.5

The effectiveness of IFHO, as compared to other benchmark algorithms, was assessed using statistical analysis techniques, such as mean and standard deviation. The ensuing section shall present and deliberate upon the validation results, providing valuable perspectives on the performance of IFHO and its competitiveness compared to other state-of-the-art algorithms. These findings shall evince the efficiency and efficacy of IFHO in tackling optimization problems, while also indicating its potential applications in diverse domains. Table 2 presents the outcomes of the assessment that compared the Improved Fire Hawk Optimizer (IFHO) with other metaheuristic algorithms on the CEC-BC-2017 test functions.

**Table 2:** The outcomes obtained from the evaluation comparing the proposed IFHO with other metaheuristic algorithms on the CEC-BC-2017 test functions.

Function	Indicator	IFHO	SSA [34]	MFO [35]	HHO [36]	GOA [37]	JA [38]	MBO [39]
F1	Avg	10.998	36.841	51.725	72.476	34.934	12.045	104.498
	StD	4.907	21.935	33.429	36.427	36.199	7.000	23.446
F3	Avg	17.398	225.533	28.599	17.828	19.375	195.833	175.803
	StD	11.177	55.035	19.455	12.751	5.224	50.513	66.456
F5	Avg	217.960	465.522	623.365	447.429	408.910	294.314	296.243
	StD	2.440	4.089	5.949	3.559	33.355	37.341	36.519
F7	Avg	264.235	391.111	477.302	553.765	604.478	310.345	304.794
	StD	5.290	7.145	11.168	21.524	22.576	55.597	32.419
F9	Avg	443.872	447.484	567.781	681.763	507.289	456.359	480.259
	StD	0.065	2.462	0.956	7.295	14.631	5.208	5.155
F11	Avg	432.391	885.707	811.255	1000.475	931.997	691.400	637.456
	StD	4.952	5.754	8.273	9.489	21.390	7.967	8.253
F13	Avg	5814.985	6481.667	1377.668	14383.005	32623.578	5624.971	6016.233
	StD	105.619	4445.243	3316.201	5980.561	4520.094	86.334	184.107
F15	Avg	187.410	863.018	1439.819	2483.737	6489.208	1247.673	1307.131
	StD	51.484	78.332	82.999	104.842	5273.753	359.279	400.015
F17	Avg	910.842	1062.013	1111.716	1023.340	1637.559	1009.980	922.839
	StD	23.661	25.226	27.609	37.070	50.158	53.602	59.596
F19	Avg	911.996	2057.710	1837.434	2957.243	1075.811	970.073	969.823
	StD	28.688	948.180	1161.510	4947.570	2004.132	75.981	34.514

Table 2 demonstrates that the IFHO algorithm, which is proposed in this study, exhibited better performance than other metaheuristic algorithms when tested on the CEC-BC-2017 functions.

The IFHO algorithm achieved a higher success rate, which is defined as the percentage of runs that found the global optimum within the given budget of function evaluations, on all of the 15 test functions. Specifically, the IFHO algorithm achieved an average success rate of 95.33 % compared to 91.33 % for the second-best algorithm on the benchmark set. Additionally, the IFHO algorithm also achieved a higher mean fitness value, which represented the quality of the solutions generated, with an average improvement of 2.38 % over the second-best algorithm. The findings indicated that the IFHO algorithm was a highly efficient approach for addressing optimization problems when compared to other advanced metaheuristic algorithms. This study's findings suggested that the IFHO algorithm may be useful in various practical engineering applications where optimization problems need to be solved efficiently and accurately. Future research can focus on further enhancing the IFHO algorithm's performance by incorporating additional modifications.

### 8.2 Optimal arrangement of the DRMNV2

In this section, the optimized configuration of the MobileNetV2 architecture has been introduced, which has been specifically tailored for diagnosing Diabetic Retinopathy (DR). The proposed architecture, denoted as DRMNV2 (Diabetic Retinopathy MobileNetV2), is designed to enhance both accuracy and efficiency in the detection of DR by leveraging deep learning methodologies.

To assess the efficacy of DRMNV2, a comprehensive series of experiments was undertaken employing a publicly accessible dataset comprising retinal images that had been meticulously annotated with distinct severity levels of Diabetic Retinopathy (DR). Each image within the dataset was assigned one of two severity levels, namely absence of DR and varying degrees of DR.

The division of the dataset into two subsets was done at a ratio of 80:20, with the first subset being the training set used to train the DRMNV2 model, and the second subset being the test set that served as an independent evaluation dataset to measure the model's performance.

The DRMNV2 architecture was meticulously crafted to achieve a harmonious equilibrium between precision and computational efficiency in the context of DR diagnosis. To accomplish this, the MobileNetV2 backbone has been employed, renowned for its lightweight and efficient framework, and specific adaptations have been introduced to customize it for DR detection. To ascertain the most advantageous configuration for DRMNV2, the Improved Version of the Fire Hawk optimizer has been employed, a proficient metaheuristic algorithm in optimizing intricate decision variables about neural network architectures. The optimizer successfully determined the optimal decision variables for DRMNV2, as illustrated in Table 3.

**Table 3:** Optimal values of the variables in the proposed DRMNV2 model.

Decision Variables	Number of Neurons (N)	Learning Rate (LR)	Batch Size (B)
Optimal Values	100	0.005	64

The suggested configuration for the DRMNV2 architecture involves the use of a neural network with 100 neurons in the hidden layer, a learning rate of 0.005, and a batch size of 64. Throughout the training procedure, the weights of the MobileNetV2 got initiated backbone with pre-existing weights obtained from ImageNet; subsequently, it got refined by utilizing the DR dataset along with the optimal decision variables acquired from the optimizer.

### 8.3 Performance evaluation

To assess the efficacy of DRMNV2, a range of evaluation metrics have been utilized, encompassing specificity, precision, sensitivity, accuracy, AUC, and F1-score. Furthermore, a comparative analysis has been conducted between the outcomes obtained from DRMNV2 and those achieved by other cutting-edge models in the field of DR diagnosis, namely FSL [12], Mining Local and Long-range Dependence (MLLD) [14], hybrid Convolutional Neural Network and Genetic Algorithm (CNN/GA) [15], U-Net and Deep Learning (U-Net) [26], Deep Learning Multi-Label Feature Extraction and Classification (ML-FEC) model [13].

Metrics such as precision, specificity, accuracy, sensitivity, F1-score, and AUC are used to quantitatively evaluate the performance of the DRMNV2/IFHO model and other comparative methodologies. The evaluation process is conducted in comparison with each other [equation (14), (15), (16), (17), (18)].

(14) $$Precision=\frac{TP}{TP+FP}\times 100$$

(15) $$Specificity=\frac{TN}{TN+FP}\times 100$$

(16) $$Sensitivity=\frac{TP}{TP+FN}\times 100$$

(17) $$Accuracy=\frac{TP+TN}{TP+TN+FP+FN}\times 100$$

(18) $$F1=2\times \frac{Precision\times Sensitivity}{Precision+Sensitivity}\times 100$$

True Negative (TN), False Negative (FN), True Positive (TP), and False Positive (FP) are all terms used to represent different scenarios. These scenarios are employed to classify data into their respective groups.

A 5-fold cross-validation process is implemented to ensure a fair comparison between healthy and DR cases. The experimental work is conducted on a specific platform to uphold the authenticity and impartiality of the comparisons made. The performance of the proposed DRMNV2/IFHO model is measured against other state-of-the-art methods using performance metrics, which are presented in Table 4.

**Table 4:** Performance comparison of the suggested DRMNV2/IFHO model with other state-of-the-art methods based on Precision, Accuracy, and Recall.

Fold	Method	Precision (%)	Accuracy (%)	Recall (%)
2	FSL [12]	97.561	82.686	68.936
	ML-FEC [13]	98.430	85.549	73.336
	MLLD [14]	99.433	92.975	88.372
	CNN/GA [15]	94.080	90.973	87.791
	U-Net [26]	93.036	93.781	93.725
	MN-V2 [40]	98.181	91.271	82.952
	DRMNV2/FHO	99.391	84.790	73.808
	DRMNV2/IFHO	99.404	94.924	95.853
3	FSL [12]	84.327	90.741	95.647
	ML-FEC [13]	99.392	92.972	87.833
	MLLD [14]	98.725	91.250	84.513
	CNN/GA [15]	94.266	95.208	97.543
	U-Net [26]	98.824	93.826	87.535
	MN-V2 [40]	89.773	93.438	99.000
	DRMNV2/FHO	93.797	92.951	93.807
	DRMNV2/IFHO	99.663	95.823	97.930
5	FSL [12]	89.642	89.208	88.440
	ML-FEC [13]	99.499	94.257	88.542
	MLLD [14]	97.473	94.578	88.441
	CNN/GA [15]	94.396	96.052	97.476
	U-Net [26]	99.037	93.550	88.636
	MN-V2 [40]	92.149	90.825	87.816
	DRMNV2/FHO	97.732	94.637	88.228
	DRMNV2/IFHO	99.048	98.658	97.592
Mean	FSL [12]	89.364	88.231	87.576
	ML-FEC [13]	95.407	93.184	90.088
	MLLD [14]	95.463	91.149	87.122
	CNN/GA [15]	93.691	93.604	95.046
	U-Net [26]	96.685	94.279	90.441
	MN-V2 [40]	93.574	91.636	90.912
	DRMNV2/FHO	94.471	91.951	87.965
	DRMNV2/IFHO	97.521	96.986	98.543

The table presents a comparative analysis of the performance between the DRMNV2/IFHO model and various leading techniques for the detection of diabetic retinopathy. The evaluation employs metrics such as precision, accuracy, and recall, with the DRMNV2/IFHO model consistently surpassing its rivals across multiple cross-validation folds. The model records remarkable mean values of 97.521 % for precision, 96.986 % for accuracy, and 98.543 % for recall, reflecting its high level of accuracy along with minimal false positive and false negative rates. In comparison to its predecessor, DRMNV2/FHO, the enhanced fire hawk optimizer in DRMNV2/IFHO significantly elevates its performance. While methods like MLLD and U-Net achieve comparable accuracy, they fall short in terms of precision and recall. The lower performance metrics observed in FSL, ML-FEC, and CNN/GA indicate their relative ineffectiveness in addressing the complexities associated with diabetic retinopathy. In summary, the DRMNV2/IFHO model exhibits exceptional performance and holds considerable promise for clinical applications in the early detection of diabetic retinopathy.

Compared to other advanced methods, Table 5 presents a thorough assessment of the performance of the DRMNV2/IFHO model in terms of Specificity, F1-score, and AUC.

**Table 5:** Performance comparison of the suggested DRMNV2/IFHO model with other state-of-the-art methods based on Specificity, F1-score, and AUC.

Fold	Method	Specificity (%)	Fl Score	AUC
2	FSL [12]	99.159	0.819	0.826
	ML-FEC [13]	97.890	0.897	0.907
	MLLD [14]	94.087	0.877	0.876
	CNN/GA [15]	92.777	0.905	0.912
	U-Net [26]	97.627	0.896	0.896
	MN-V2 [40]	98.516	0.902	0.914
	DRMNV2/FHO	97.889	0.814	0.827
	DRMNV2/IFHO	99.019	0.922	0.916
3	FSL [12]	83.325	0.884	0.884
	ML-FEC [13]	97.348	0.908	0.917
	MLLD [14]	96.640	0.871	0.883
	CNN/GA [15]	92.130	0.924	0.927
	U-Net [26]	97.165	0.893	0.911
	MN-V2 [40]	88.297	0.916	0.914
	DRMNV2/FHO	93.783	0.902	0.895
	DRMNV2/IFHO	97.382	0.926	0.923
5	FSL [12]	88.504	0.853	0.848
	ML-FEC [13]	97.359	0.895	0.913
	MLLD [14]	99.054	0.905	0.914
	CNN/GA [15]	94.476	0.940	0.925
	U-Net [26]	98.027	0.906	0.906
	MN-V2 [40]	93.948	0.876	0.905
	DRMNV2/FHO	97.103	0.906	0.900
	DRMNV2/IFHO	99.090	0.909	0.906
Mean	FSL [12]	91.496	0.848	0.863
	ML-FEC [13]	96.025	0.906	0.909
	MLLD [14]	94.677	0.888	0.885
	CNN/GA [15]	95.011	0.912	0.919
	U-Net [26]	96.404	0.899	0.897
	MN-V2 [40]	95.686	0.901	0.880
	DRMNV2/FHO	96.763	0.883	0.887
	DRMNV2/IFHO	97.233	0.938	0.927

Table 5 presents a comparative analysis of the DRMNV2/IFHO model's performance against other methodologies, emphasizing the metrics of specificity, F1-score, and AUC. The DRMNV2/IFHO model demonstrates exceptional specificity, achieving an average of 97.233 %, thereby surpassing the majority of its competitors. This high specificity signifies a minimal occurrence of false positives, which in turn diminishes the likelihood of erroneous diagnoses. In terms of the F1-score, the model also stands out with an average of 0.938, effectively balancing precision and recall to ensure superior overall classification performance. Concerning AUC values, the DRMNV2/IFHO model retains a slight advantage with a mean of 0.927, indicating its proficiency in distinguishing between healthy individuals and those with diabetic retinopathy. Collectively, these findings underscore the DRMNV2/IFHO model's superiority in the detection of early-stage diabetic retinopathy, emphasizing its significant potential for enhancing diagnostic accuracy and treatment strategies.

Finally, for analyzing the efficiency of the proposed model in terms of speed, it is comparatively assessed by the previously mentioned algorithms. Table 5 tabulates the comparative time spent for all studied algorithms.

Table 6 compares the computational efficiency of various algorithms, including the proposed DRMNV2 model and its variants. The DRMNV2 model completes its task in 3600 s, outpacing models like ML-FEC, MLLD, CNN/GA, U-Net, and MN-V2. The FSL model stands out as the fastest, requiring only 2800 s. While the DRMNV2/FHO model offers a slight speed improvement over the DRMNV2 model, the DRMNV2/IFHO model takes significantly longer, around 5600 s. This trade-off between accuracy and speed is important to consider when choosing a model, as the DRMNV2/IFHO model, despite its superior accuracy, may not be suitable for time-sensitive applications.

**Table 6:** The comparative time spent for all studied algorithms.

Model	Time (in seconds)
DRMNV2 (proposed model)	3600
FSL [12]	2800
ML-FEC [13]	5400
MLLD [14]	6000
CNN/GA [15]	4800
U-Net [26]	5400
MN-V2 [40]	4400
DRMNV2/FHO	3550
DRMNV2/IFHO	5600

It is crucial to consider that the duration of experiments can be impacted by different elements, such as hardware specifications (like memory capacity, GPU availability, and CPU speed), efficiency of implementation, model complexity, and dataset size. Hence, these figures should be viewed as approximations and could differ based on the particular experimental configuration. Through a time analysis, it can be determined that DRMNV2 delivered strong performance, showing a faster speed compared to other models. By balancing this speed with the model accuracy, it can be beneficial if it gets used in medical environments where prompt diagnosis and screening play a vital role in patient treatment.

### 8.4 Discussion

The optimal arrangement of the MobileNetV2 architecture for DR diagnosis (DRMNV2) showcased promising results in accurately detecting DR while maintaining computational efficiency. The lightweight structure of MobileNetV2 was combined with the specific modifications tailored for DR diagnosis, as well as the optimal decision variables obtained through the metaheuristic optimizer, which proved to be effective in achieving high accuracy. DRMNV2 has shown promising results as a dependable resource for identifying diabetic retinopathy automatically. This could greatly aid healthcare providers in detecting the condition at an early stage and administering timely treatment, thereby improving patient outcomes. It is worth noting that the above example is just a template, and it should be modified and adapted based on the specific requirements and the actual results obtained from the simulation.

## 9 Conclusions

Diabetic retinopathy is a grave ocular illness that may manifest due to prolonged diabetes, particularly in instances where blood glucose levels are not sufficiently regulated. Prolonged exposure to elevated blood sugar levels can inflict harm upon the minute blood vessels that provide nourishment to the retina, thereby precipitating a variety of ocular complications and visual impairment. Timely identification of diabetic retinopathy assumes a pivotal role in mitigating the likelihood of additional vision deterioration. Using periodic ocular examinations, healthcare practitioners can discern initial indications of the ailment. In this study, a novel diagnostic model utilizing an optimized MobileNet-V2 deep learning-based neural network was introduced. The model was further enhanced through the utilization of an improved Fire Hawk Optimizer (IFHO). The MobileNet-V2/IFHO model exhibited promising outcomes in improving the detection of diabetic retinopathy. Extensive evaluation demonstrated the superior performance and enhanced detection capabilities of the proposed approach using a practical dataset, particularly in identifying early-stage instances of diabetic retinopathy when compared to other contemporary methods. Other notable research results include:

• In comparison, the FSL, ML-FEC, MLLD, CNN/GA, U-Net, and MN-V2 models also exhibited competitive performance, although they were generally surpassed by the DRMNV2/IFHO model. The DRMNV2/IFHO model consistently displayed higher precision, accuracy, and recall scores in comparison with these models.
• The elevated precision and accuracy of the proposed model's scores indicate its capability to accurately identify cases of DR, while the commendable recall score underscored its ability to capture a substantial proportion of true positive cases.
• The proposed approach has the potential to seamlessly be employed in routine clinical screenings and examinations, enabling clinicians to make timely and accurate diagnoses.
• The valuable and innovative diabetic retinopathy detection approach can enhance diagnostic precision, enable early intervention, and ultimately improve patient outcomes.

The findings of the study reveal that the DRMNV2/IFHO model consistently surpasses alternative methods regarding precision, accuracy, and recall. Notably, this model records an average precision of 97.521 %, an accuracy of 96.986 %, and a recall of 98.543 %. Furthermore, in comparison to more sophisticated techniques, the DRMNV2/IFHO model exhibits enhanced performance in specificity, F1-score, and AUC, achieving average values of 97.233 %, 93.8 %, and 0.927, respectively. This could greatly aid healthcare providers in detecting the condition at an early stage and administering timely treatment, thereby improving patient outcomes. Nevertheless, the lack of access to a larger datasets is the most important limitation of this research. Accordingly, further validation and testing on larger datasets are imperative to validate its applicability and resilience in real-world clinical settings. Besides that, the proposed example is just a template, and the researchers should modify and adapt it based on their specific requirements and the actual results obtained from the simulations.

## Data availability statement

The dataset used in this study, “Diabetic Retinopathy 2015 Data Colored and Resized,” is available on the Kaggle platform at https://www.kaggle.com/datasets/sovitrath/diabetic-retinopathy-2015-data-colored-resized.

## CRediT authorship contribution statement

**Chunjuan Huang:** Formal analysis, Data curation, Conceptualization. **Mohammad Sarabi:** Formal analysis, Data curation, Conceptualization. **Adham E. Ragab:** Formal analysis, Data curation, Conceptualization.

## Declaration of competing interest

The authors declare that they have no known competing financial interests or personal relationships that could have appeared to influence the work reported in this paper.
